# The Effect of Alendronate Loaded Biphasic Calcium Phosphate Scaffolds on Bone Regeneration in a Rat Tibial Defect Model

**DOI:** 10.3390/ijms161125982

**Published:** 2015-11-06

**Authors:** Kwang-Won Park, Young-Pil Yun, Sung Eun Kim, Hae-Ryong Song

**Affiliations:** Institute for Rare Diseases and Department of Orthopaedic Surgery, Korea University Medical Center, Guro Hospital, 148 Gurodong-ro, Guro-gu, Seoul 152-703, Korea; kwpark77@gmail.com (K.-W.P.); ofeel0479@korea.ac.kr (Y.-P.Y.); sekim10@korea.ac.kr (S.E.K.)

**Keywords:** alendronate, biphasic calcium phosphate, scaffold, bone formation

## Abstract

This study investigated the effect of alendronate (Aln) released from biphasic calcium phosphate (BCP) scaffolds. We evaluated the *in vitro* osteogenic differentiation of Aln/BCP scaffolds using MG-63 cells and the *in vivo* bone regenerative capability of Aln/BCP scaffolds using a rat tibial defect model with radiography, micro-computed tomography (CT), and histological examination. *In vitro* studies included the surface morphology of BCP and Aln-loaded BCP scaffolds visualized using field-emission scanning electron microscope, release kinetics of Aln from BCP scaffolds, alkaline phosphatase (ALP) activity, calcium deposition, and gene expression. The *in vitro* studies showed that sustained release of Aln from the BCP scaffolds consisted of porous microstructures, and revealed that MG-63 cells cultured on Aln-loaded BCP scaffolds showed significantly increased ALP activity, calcium deposition, and gene expression compared to cells cultured on BCP scaffolds. The *in vivo* studies using radiograph and histology examination revealed abundant callus formation and bone maturation at the site in the Aln/BCP groups compared to the control group. However, solid bony bridge formation was not observed at plain radiographs until 8 weeks. Micro-CT analysis revealed that bone mineral density and bone formation volume were increased over time in an Aln concentration-dependent manner. These results suggested that Aln/BCP scaffolds have the potential for controlling the release of Aln and enhance bone formation and mineralization.

## 1. Introduction

With recent developments in tissue engineering and regenerative medicine, bone graft substitutes are becoming standard for reconstructing large skeletal defects after trauma, tumor, and joint surgery. The optimal bone graft substitute should offer osteoconduction, osteoinduction, and osteogenesis [1]. Various type of tissue engineered scaffolds have been developed [2]; among them, bioceramics, such as calcium phosphates, are attractive owing to their degradability, bioactivity, biocompatibility, and osteoconductivity [3]. The most widely investigated calcium phosphates are hydroxyapatite (HAp) and beta-tricalcium phosphate (β-TCP), and mixtures thereof, referred to as biphasic calcium phosphate (BCP) [4].

BCP is similar to the mineral phase of natural bone and has been approved by the Food and Drug Administration (FDA) for many applications in the fields of dental and orthopedic surgery [5,6,7]. BCP-based scaffolds have several advantages including bioactivity, partial biodegradation, and resorption a few months after implantation. Moreover, these materials enhance osteoblast proliferation, as well as osteogenic differentiation [4,8,9]. Nevertheless, although BCP scaffolds have efficient bone-forming capacity, they often do not stimulate adequate revascularization, cellular reconstitution, or osteogenesis necessary for successful biointegration. Therefore, BCP scaffolds containing osteoinductive materials are required for more effective bone regeneration.

Bisphosphonates are a class of drugs usually used in treating bone disorders such as osteoporosis, Paget’s disease, fibrous dysplasia, hypercalcemia of malignancy, and inflammation related bone loss [10,11,12]. Among the bisphosphonates, alendronate (Aln) is one of the most commonly used drugs that effectively inhibit bone resorption. Recent studies have reported that Aln induces osteogenic differentiation of osteoblasts, bone marrow mesenchymal stem cells, and adipose-derived stem cells [13,14,15]. However, Aln easily dissolved in aqueous conditions during fabrication because of its high hydrophilicity. Therefore, better-controlled drug release systems capable of achieving efficient osteogenesis are urgently needed; in response, researchers have tried to find proper carriers that can provide an osteoconductive matrix and impart handling properties required for implantation at the repair site in order to improve Aln loading and avoid side effects [16,17,18].

There have been several attempts to develop calcium phosphate based scaffolds as delivery systems for bisphosphonate [19,20,21]. However, clinical use of calcium phosphate based scaffolds had been limited due to several drawbacks—such as the difficulty in molding [22], unspecified irregular shapes and sizes [21], limited drug loading content [19], and burst release upon administration [17].

BCP scaffolds consisted with unique dual pore structure, which enables increased biological affinity to neovascularization, osteoblast cell migration, and osteoblast cell integration. Furthermore, BCP scaffolds could provide more controlled release of Aln due to its dual pore structure.

In this study, we prepared BCP scaffolds that maintained Aln concentration at the repair site long enough to allow bone-forming cells to migrate to the defect site, proliferate, and differentiate in response to Aln. The characteristics of BCP scaffolds containing Aln were analyzed, and we tried to determine that (1) Aln/BCP scaffolds could facilitate osteogenic differentiation of bone-forming cells *in vitro* and (2) the *in vivo* bone regenerative capability of Aln/BCP scaffolds using a rat tibial defect model.

## 2. Results

### 2.1. Characterization of Biphasic Calcium Phosphate (BCP) and Modified BCP Scaffolds

**Figure 1:**
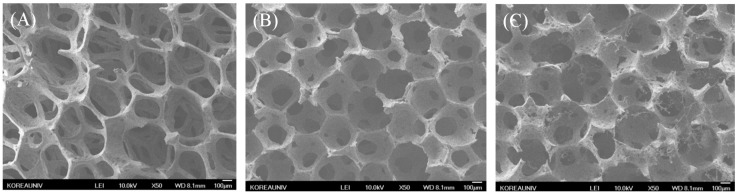

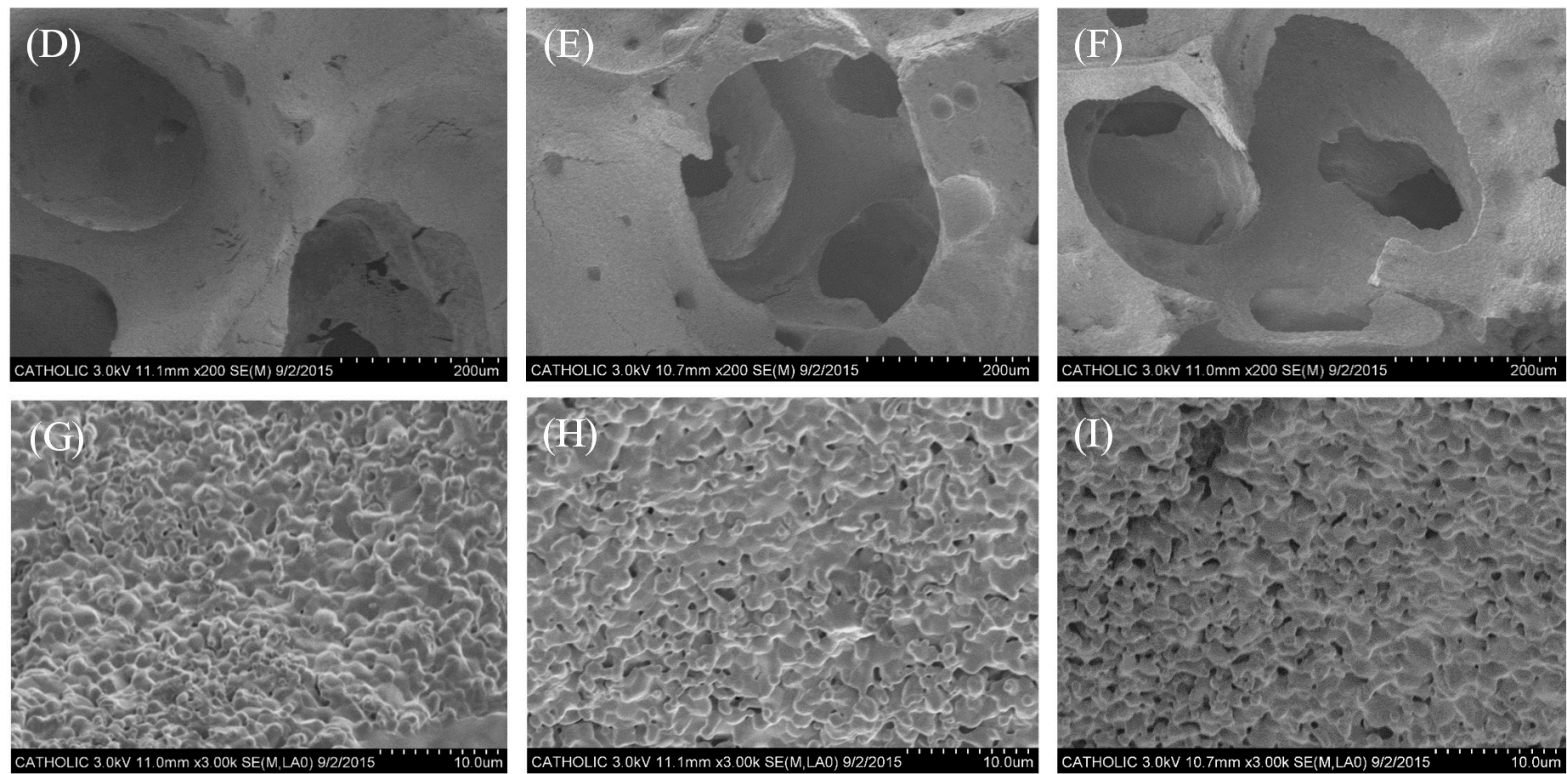
(**A**–**I**) 50× magnification of scanning electron microscope (SEM) images of (**A**) BCP, (**B**) Aln (1 mg)/BCP and (**C**) Aln (5 mg)/BCP. The scaffolds in each groups showed open pore microstructures and round-shaped pores with diameters ranging 100 to 300 μm; The characteristic dual pores were visualized in 200× magnification images of (**D**) BCP, (**E**) Aln (1 mg)/BCP and (**F**) Aln (5 mg)/BCP; Micropores were visualized at 3000× magnification images of (**G**) BCP, (**H**) Aln (1 mg)/BCP and (**I**) Aln (5 mg)/BCP.

Field-emission scanning electron microscopy (FE-SEM) of the morphologies of BCP, Aln (1 mg)/BCP, and Aln (5 mg)/BCP scaffolds showed open dual pore microstructures and round-shaped pores with diameters ranging 100 to 300 μm (Figure 1A–I). The surface elemental composition of the BCP scaffold consisted of carbon, oxygen, phosphorus, and calcium. In contrast, Aln-containing BCP scaffolds revealed the presence of carbon, oxygen phosphorous, calcium, and nitrogen (Table 1). This result indicates that Aln was successfully anchored on BCP scaffold. The average loading amount of Aln on BCP scaffold was 786.28 ± 6.68 µg in Aln (1 mg)/BCP scaffold and 3638.49 ± 7.12 µg in Aln (5 mg)/BCP scaffold, and their loading efficiency was 78.63% ± 0.67% and 72.77% ± 0.14%, respectively (Table 2).

**Table 1:** Surface elemental composition of Biphasic Calcium Phosphate (BCP) and Alendronate (Aln)/BCP scaffolds.

Samples	Elements					
	C (%)	O (%)	P (%)	Ca (%)	N (%)	Total (%)
BCP	22.20	53.19	12.32	12.29	0	100
Aln (1 mg)/BCP	9.87	45.66	11.76	21.68	11.03	100
Aln (5 mg)/BCP	16.55	38.75	10.36	22.87	11.47	100

BCP: Biphasic calcium phosphate.

**Table 2:** Loaded amount of Aln on BCP scaffolds.

Samples	Loading Amount (µg)	Loading Efficiency (%)
Aln (1 mg)/BCP	786.28 ± 6.68	78.63 ± 0.67
Aln (5 mg)/BCP	3638.49 ± 7.12	72.77 ± 0.14

BCP: Biphasic calcium phosphate.

### 2.2. Release Kinetics of Alendronate (Aln) from BCP Scaffold

**Figure 2:**
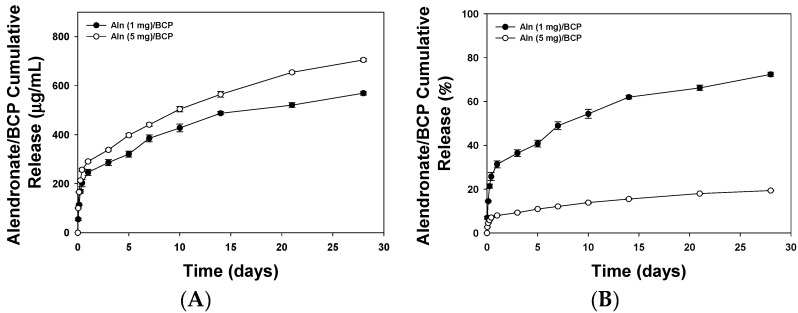
(**A**) Cumulative *in vitro* release profile of Aln from Aln (1 mg)/BCP and Aln (5 mg)/BCP scaffolds, respectively. The amounts of Aln released from BCP scaffold were similar in spite of different concentrations; and (**B**) the percentage of cumulative *in vitro* release profile of Aln shows different releasing pattern depending on their concentration. On the first day, 31.33% ± 1.58% of Aln was released from Aln (1 mg)/BCP scaffold, whereas 7.99% ± 0.08% of Aln was released from Aln (5 mg)/BCP scaffold. On the 28th day, 72.42% ± 1.01% of Aln was released from Aln (1 mg)/BCP scaffold, whereas 19.36% ± 0.16% of Aln was released from Aln (5 mg)/BCP scaffold.

Figure 2A shows the release profiles of Aln from Aln (1 mg)/BCP and Aln (5 mg)/BCP scaffolds. Sustained release of Aln from Aln (1 mg)/BCP and Aln (5 mg)/BCP scaffolds was observed for up to 28 days. On the first day, 196.36 ± 0.02 μg and 290.77 ± 0.01 μg of Aln were released from Aln (1 mg)/BCP and Aln (5 mg)/BCP scaffolds, respectively. A total of 569.42 ± 0.03 μg and 705.34 ± 0.02 μg of Aln were released from Aln (1 mg)/BCP and Aln (5 mg)/BCP scaffolds, respectively. However, the proportion of released Aln was different depending on their concentration of Aln in BCP scaffolds. High concentration of Aln (5 mg)/BCP scaffold shows more sustained release of Aln during the test up to 28 days. During 28 days, more than 70% (72.42 ± 1.01) of Aln was released from Aln (1 mg)/BCP scaffold, whereas less than 20% (19.36 ± 0.16) of Aln was released from Aln (5 mg)/BCP scaffold (Figure 2B).

### 2.3. Alkaline Phosphatase (ALP) Activity and Calcium Contents

**Figure 3:**
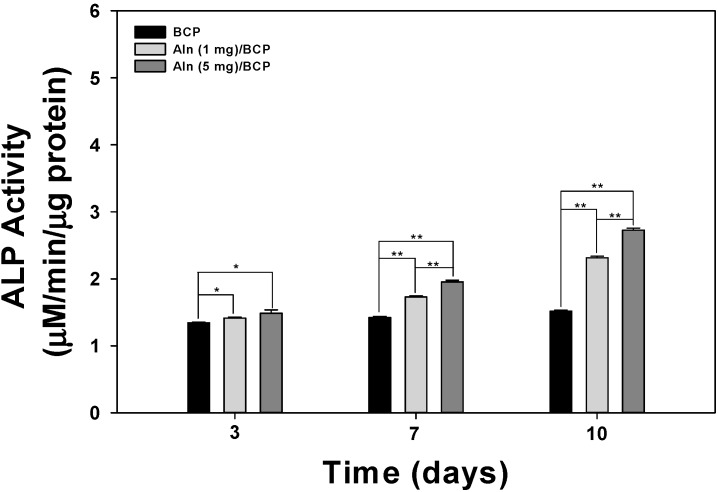
Alkaline phosphatase (ALP) activity of MG-63 cells cultured on BCP, Aln (1 mg)/BCP, and Aln (5 mg)/BCP after three, seven, and 10 days of incubation. The error bars represent mean ± SD (*n* = 5). (* *p* < 0.05 and ** *p* < 0.01).

**Figure 4:**
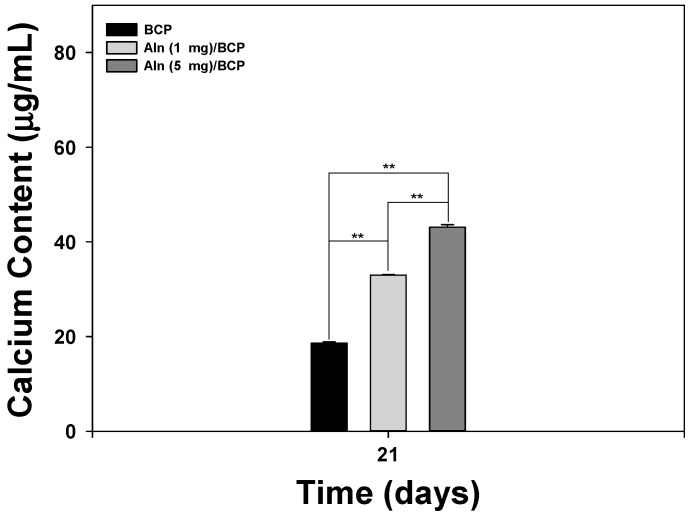
Calcium deposition by MG-63 cells cultured on BCP, Aln (1 mg)/BCP, and Aln (5 mg)/BCP after 21 days of incubation. The error bars represent mean ± SD (*n* = 5). (** *p* < 0.01).

Figure 3 shows the ALP activity of MG-63 cells cultured on BCP, Aln (1 mg)/BCP, and Aln (5 mg)/BCP scaffolds at three, seven, and 10 days. The ALP activity of MG-63 cells grown on all scaffolds gradually increased with incubation times up to 10 days. The ALP activity of MG-63 cells cultured on Aln-containing BCP scaffolds was higher compared with those cultured on BCP scaffolds at three days. At days seven and 10, the ALP activity of MG-63 cells cultured on Aln (1 mg)/BCP and Aln (5 mg)/BCP scaffolds was significantly different than those cultured on BCP scaffolds (** *p* < 0.01). In addition, there was also a significant difference in the ALP activity of MG-63 cells cultured on Aln (5 mg)/BCP scaffolds compared with Aln (1 mg)/BCP (** *p* < 0.01). Calcium deposition was measured after MG-63 cells were cultured for 21 days on BCP, Aln (1 mg)/BCP, and Aln (5 mg)/BCP scaffolds (Figure 4). There was a statistically significant difference in the amount of calcium deposited on MG-63 cells between the Aln-containing BCP and BCP scaffolds (** *p* < 0.01).

### 2.4. Gene Expression

**Figure 5:**
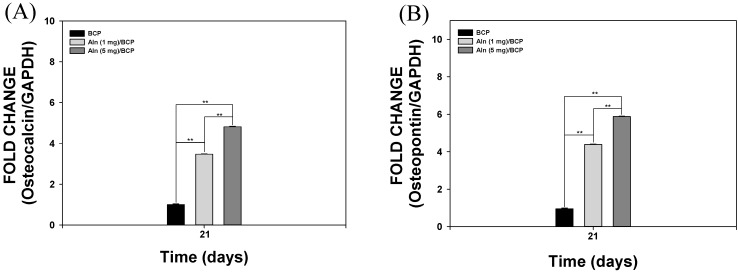
Real-time PCR analysis for (**A**) osteocalcin and (**B**) osteopontin expression of MG-63 cells cultured on BCP, Aln (1 mg)/BCP and Aln (5 mg)/BCP after seven and 21 days of incubation. The error bars represent mean ± SD (*n* = 5). (** *p* < 0.01).

mRNA expression of osteocalcin (OCN) and osteopontin (OPN) in MG-63 cells cultured on BCP, Aln (1 mg)/BCP, and Aln (5 mg)/BCP scaffolds was characterized by real-time PCR after culturing for 21 days (Figure 5A,B). The mRNA expression of OCN and OPN were significantly higher in MG-63 cells grown on Aln-containing BCP scaffolds compared with those grown on BCP scaffolds after 21 days of culture (** *p* < 0.01). The mRNA expression levels of OCN and OPN in MG-63 cells cultured on Aln (5 mg)/BCP scaffolds were markedly higher compared to those cultured on Aln (1 mg)/BCP scaffolds after 21 days of culture (** *p* < 0.01).

### 2.5. Bone Formation Evaluation

**Figure 6:**
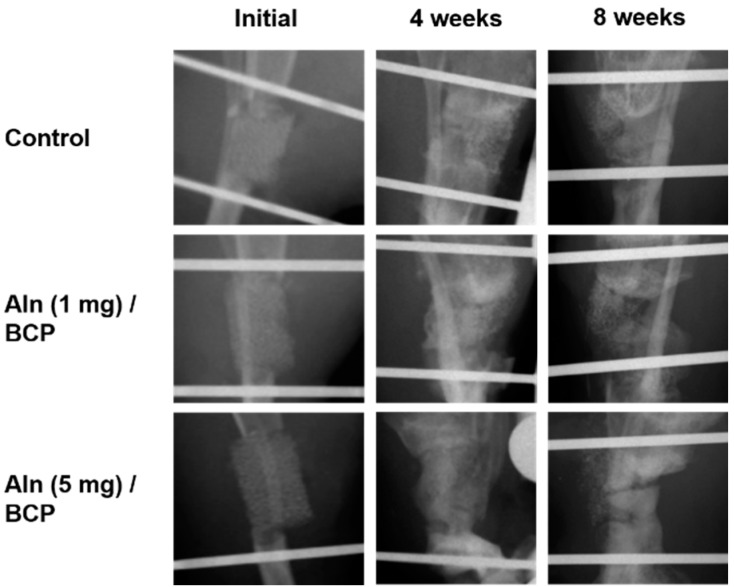
Plain radiographs of rat tibial defect model. The sharp margin of the osteotomy sites were disappeared with laps of time at four and eight weeks in all specimens. More bone formation and high radio-opaque consolidation of the defect areas were observed at eight weeks in Aln (5 mg)/BCP scaffold model specimens. However, no solid bony bridging was observed in any of the all three groups, the Aln/BCP groups showed relatively abundant callus formations compared to the control group.

As shown in Figure 6, sharp margin of the osteotomy sites were disappeared with laps of time at four and eight weeks in all specimens. However, those blunt margins were still remained until eight weeks in all specimens, even though more bone formation and high radio-opaque consolidation of the defect areas were observed at eight weeks in Aln (5 mg)/BCP scaffold model specimens. Moreover, no solid bony bridging was observed in any of the all three groups, the Aln/BCP groups showed abundant callus formations compared to the control group.

**Figure 7:**
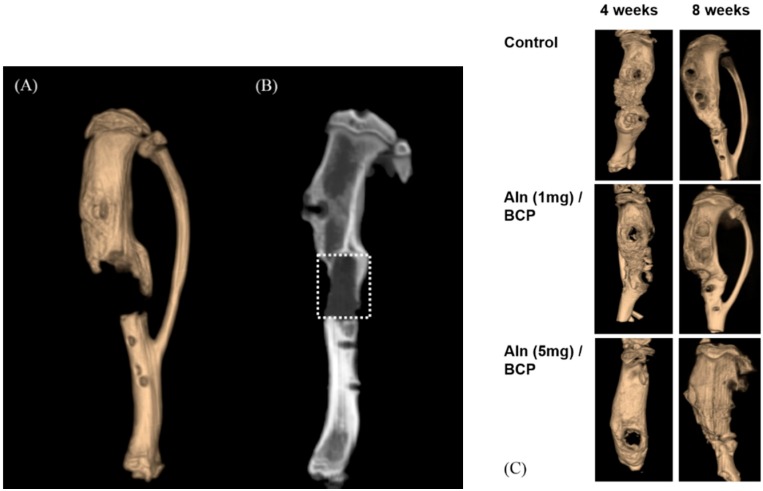
(**A**–**C**) Micro-computed tomography (CT) analysis was performed for analyzing the amount of bone formation at the fourth and eight weeks post operation. (**A**) Three-dimensional micro-CT image shows incomplete bony bridge formation at the defect site; (**B**) the amount of bone formation was evaluated within boundaries of the newly formed bone (white dotted square) using bone mineral density and bone formation volume (%BV); (**C**) 3-dimentional micro-CT images of three groups at the eighth week shows relatively consolidated new bone formation compared to the images taken at the fourth week post operation.

**Figure 8:**
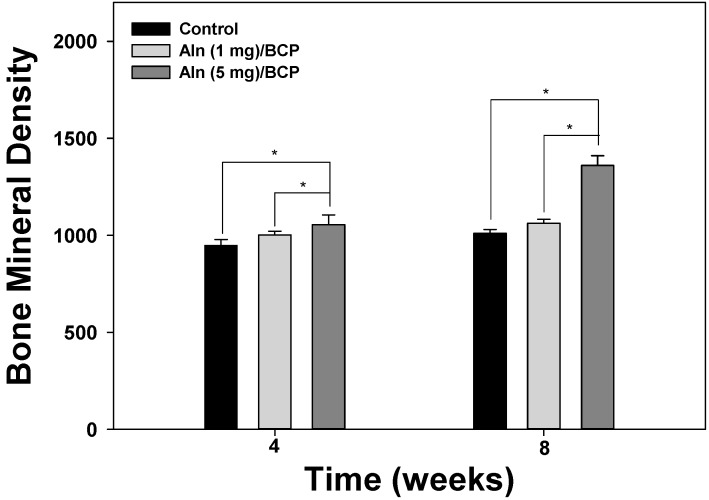
Bone mineral density at four and eight weeks after implantation. The error bars represent mean ± SD (*n* = 5). (* *p* < 0.05).

**Figure 9:**
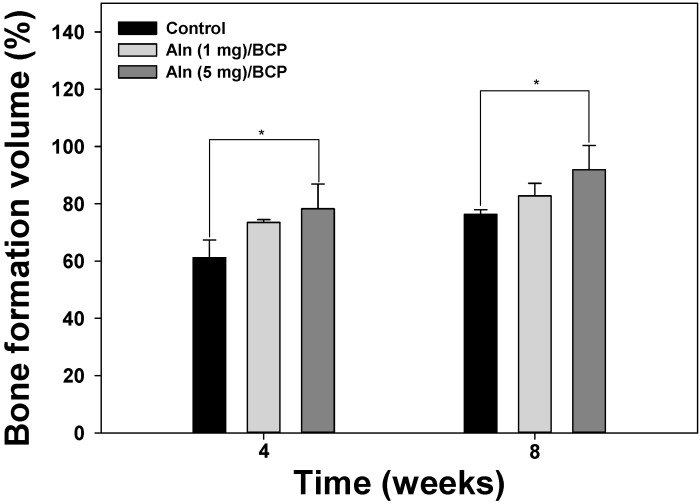
Bone formation volume (%) at four and eight weeks after implantation. The error bars represent mean ± SD (*n* = 5). (* *p* < 0.05).

Micro-CT was used to evaluate the amount of bone formation at four and eight weeks post operation (Figure 7A–C). Three-dimentional micro-CT images of three groups at the eight week show relatively consolidated new bone formation compared to the images taken at the fourth week post operation. The bone mineral density increased over time in an Aln-concentration-dependent manner (Figure 8). Bone formation volume (%BV) was calculated as the percentage of new bone area in the total augmented area, including all tissues within the boundaries of the newly formed bone. Bone formation volume was significantly increased at four and eight weeks in Aln (5 mg)/BCP scaffold model specimens compared to the control group (Figure 9).

### 2.6. Histological Evaluation

**Figure 10:**
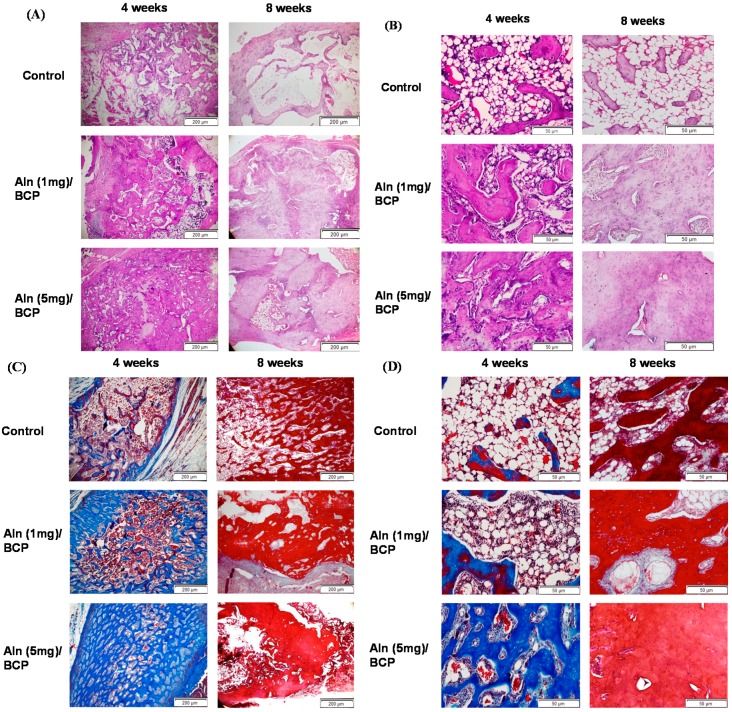
(**A**–**D**) Representative sections of (**A**) hematoxylin and eosin staining and (**C**) Goldner’s trichrome staining four and eight weeks after implantation (40× magnification). Abundant surrounding fibrous tissue formation and woven bone formation at the defect are visible in the Aln (5 mg)/BCP scaffold. Similar findings were observed on high power field (200× magnification) (**B**,**D**).

Histological analysis confirmed that Aln/BCP scaffolds showed improved new bone formation *in vivo*. H and E staining showed scanty bone formation at 40× and 200× magnification in the control group, whereas abundant osteoid tissues were observed at eight weeks in Aln (5 mg)/BCP scaffolds group specimen (Figure 10A,B).

Masson’s trichrome staining showed active new bone formation in specimens four weeks post-operation at 40× and 200× magnification, whereas scanty bone formation was visible in the control group. At eight weeks, the defect area was filled with mature bone tissue (Figure 10C,D).

## 3. Discussion

In bone tissue engineering, scaffolds should provide improved cell adhesion, proliferation, and mineralization. BCP scaffolds are considered osteoconductive bone substitutes. Osteoconductive materials provide a framework for vascular and cellular infiltration, but do not stimulate osteogenic differentiation of mesenchymal stem cells or osteoblast-like cells. For more effective bone regeneration, BCP scaffolds containing osteoinductive materials are required. Although the principal mode of action of Aln is inhibition of osteoclast function by inhibiting the mevalonate pathway of cholesterol synthesis [12], recent studies showed that local delivery of Aln by calcium phosphate scaffolds promote osteoblast differentiation and mineralization *in vitro* [23,24]. Previous studies have revealed that Aln can up-regulate mRNA expression during osteogenic differentiation *in vitro*, including that of bone morphogenetic protein 2, type I collagen, osteocalcin, and osteopontin [16,25]. Aln can also increase osteocalcin expression, mineralization, and unprenylated Rap1 in human mesenchymal stem cells [26]. Local Aln treatment enhanced proliferation and differentiation of bone-forming cells adjacent to the bone surface *in vivo* mediated through inhibition of the mevalonate pathway [27]. However, the local delivery of Aln is composed of two-step process—first, preparation of BCP scaffolds, and second, the Aln loading on BCP scaffolds. Therefore, there have been several concerns of limited drug loading content, heterogeneous drug distribution within the scaffold, uncontrolled release kinetics [19,21]. Accordingly, we tried to analyze the characteristics of BCP scaffolds containing Aln, and investigate whether MG-63 cells cultured on BCP scaffolds containing Aln had enhanced osteoblast activity compared with those cultured on BCP-only scaffolds, and performed *in vivo* study of Aln/BCP scaffolds in a rat tibial defect model.

One of the main advantages of a BCP scaffold is its unique, dual pore structure, which is comprised of macro- and micro-pores. Macro-pores enable easy neovascularization and osteoblast cell migration, while micro-pores have a high binding affinity to osteoblast cells. In our study, FE-SEM images confirmed multiple porous structures consisted with the coating of Aln on BCP scaffolds by elemental composition analysis. Increased N content was observed with increasing concentrations of Aln coated on BCP scaffolds. Moreover, Aln-coated BCP scaffolds were also evaluated by XPS K-Alpha spectroscopy to confirm the presence of Aln functional groups. The hydroxyl and P=O functional groups of Aln were observed on BCP scaffolds containing Aln, not on BCP-only scaffolds. These results indicate that Aln was successfully coated on BCP scaffolds.

In this study, the Aln release was sustained over 30 days, and the release showed relatively linear kinetics, except for a burst release during the initial 24 h (Figure 2A,B). The initial burst release might be attributed the Aln bound on the BCP scaffold surface. And sustained and slow release of Aln might be ascribed the biphasic characteristics of BCP, by the calcium phosphate mineral dissolution [17,28,29,30]. When considering the sustained release kinetics, the Aln/BCP scaffold might release Aln over several months. Therefore, this Aln/BCP scaffolds may serve as useful long-term local Aln delivery systems in bone defect model *in vivo*. However, we should not overlook the possibility of mismatch between the *in vitro* release study and *in vivo* situation. Therefore, future study using *in vivo* labeling of the Aln could be considered to know the real concentration of Aln at the defect site.

To demonstrate enhanced osteogenesis for all BCP groups, ALP activity and calcium deposition, which are important markers for early and late osteogenic differentiation of osteoblast-like cells [18,31], were analyzed. Previous studies demonstrated that synovial mesenchymal stem cells grown on Aln-loaded poly lactic-co-glycolic acid (PLGA)/HAp-sintered microspherical scaffolds and osteoblasts cultured on Aln-loaded novel PLGA/HAp microspheres have higher ALP activity compared with the control group [15,32]. More recently, Moon *et al.* [16] and Kim *et al.* [18] showed that the ALP activity and calcium deposition of MC3T3-E1 cells and MG-63 cells cultured on Aln-immobilized titanium (Ti) and Aln-eluting chitosan scaffolds, respectively, were significantly different compared to the control group. In our study, the ALP activity of MG-63 cells was evaluated after a culture period of 10 days. The ALP activity increased over the 10-day period for all BCP scaffolds. However, the activity of MG-63 cells grown on BCP scaffolds containing Aln was significantly higher than those grown on BCP-only scaffolds after three days. In addition, the ALP activity of MG-63 cells cultivated on Aln (5 mg)/BCP was significantly different from the ALP activity of MG-63 cells cultivated on Aln (1 mg)/BCP after seven days. As expected from the ALP activity data in the present study, the amount of calcium deposition on MG-63 cells grown on Aln-containing BCP scaffolds was significantly higher than those grown on BCP-only scaffolds after 21 of incubation. Taken together, these findings indicate that Aln can stimulate early and late osteogenic differentiation.

After 21 days of culture, expression of OCN and OPN genes in MG-63 cells grown on all BCP scaffolds confirmed osteogenic differentiation. Previous studies reported that OCN and OPN genes, markers of osteoblast differentiation, are up-regulated after osteoblast differentiation [33,34]. In the present study, expression of OCN and OPN genes in MG-63 cells cultured on BCP scaffolds containing Aln was markedly upregulated compared to cells cultured on BCP-only scaffolds. These results indicate that BCP scaffolds containing Aln stimulated osteogenic differentiation of MG-63 cells by releasing Aln. Particularly, Aln (5 mg)/BCP scaffolds showed significantly greater osteogenic differentiation of MG-63 cells compared to Aln (1 mg)/BCP scaffolds. Thus, BCP scaffolds containing Aln effectively stimulated osteogenic differentiation, indicating the potential use for regeneration of bone defects.

We performed *in vivo* studies to determine whether Aln/BCP scaffolds show improved bone-regenerating capabilities compared with BCP scaffolds alone using a rat tibial defect model. In our study, x-ray images and micro-CT analysis revealed that bone growth of Aln/BCP scaffolds increased with increased Aln content. However, we could not determine the quality and mechanical strength of regenerate bone, and patterns of biodegradation of BCP scaffolds. Moreover, there were no significant differences in bone mineral density and bone formation volume between the lower-dose Aln (1 mg)/BCP scaffold group and the control group. It is hard to determine that the results arise from long term cumulative effect of Aln or dose dependent effect of Aln. In histological analysis revealed abundant woven bone formation with Aln (5 mg)/BCP scaffolds four weeks after implantation, and the majority of the woven bone had been converted into mature bone at eight weeks. These findings suggested that Aln/BCP scaffolds had good potential of osteoinduction, enhancing osteogenesis, and bone regeneration.

Although Aln has been widely used to treat various conditions [10,11,12], there have been concerns about the uncoupling effect [35,36,37]. Systemic administration and long term usage of Aln are likely related to the potent over-suppression of bone turnover and inhibitory effects on osteogenesis [37,38]. In this study, local delivery of Aln using BCP scaffolds did not show any negative effect on bone regeneration up to eight weeks after implantation in the rat tibia defect model. However, the Aln/BCP scaffold did result in a significant difference in bone formation over an eight-week period with higher dose of Aln (5 mg). The results of this study suggest that *in vivo* applications of Aln/BCP scaffolds is efficacious for bone regeneration.

This study has several limitations. First, cumulative effect of Aln should be investigated more than eight weeks after implantation. Previous clinical studies using BCP reported that BCP granules last longer than two years and did not completely remodel [39,40]. The locally delivered Aln might affect remodeling of newly regenerated bone as well as osteoclast-related material absorption. Radiographic evaluation should be considered to test the length of bony consolidation, remodeling, and material absorption. Second, biomechanical study of regenerated bone should be performed. The quality and structural stability of the regenerated bone should be evaluated thoroughly.

## 4. Experimental Section

### 4.1. Materials

Biphasic calcium phosphate (BCP, HAp = 60%, TCP = 40%) scaffolds were kindly donated by OssGen Corporation (Gyeongbuk, Korea). Aln was obtained from Samjin Pharmaceutical Corporation (Seoul, Korea). Dulbecco’s Modified Eagle’s Medium (DMEM), phosphate buffer saline (PBS), fetal bovine serum (FBS), and 1% antibiotics (100 U/mM penicillin and 0.1 mg/mL streptomycin) were purchased from Life Technologies Corporation (Grand Island, NY, USA). 2-(*N*-Morpholino) ethanesulfonic acid (MES) was supplied from Sigma Chemical Co. (St. Louis, MO, USA). MG-63 cells (human osteosarcoma cell line) were obtained from Korea Cell Line Bank (KCLB No. 21427, Seoul, Korea).

### 4.2. Preparation of BCP Scaffold Coated with Alendronate (Aln)

Two different Aln concentrations (1 mg or 5 mg) were dissolved in 0.1 M MES buffer (pH 5.6), respectively. The concentrations of Aln were determined based on our previous studies [18,41]. BCP scaffolds were then immersed in 0.1 M MES buffer dissolved Aln and allowed to react for 24 h with gentle shaking. Aln-coated BCP scaffolds were collected, washed with distilled water, and vacuum-dried for one day.

### 4.3. Characterization of BCP and Aln-Coated BCP

To demonstrate the surface morphologies and elemental compositions of BCP, Aln (1 mg)/BCP, and Aln (5 mg)/BCP, field-emission scanning electron microscopy (FE-SEM, JSM-6700F, JEOL, Tokyo, Japan) was performed at the College of Health Science on Korea University. The samples were coated with platinum using a sputter-coater (Cressington 108; Cressington Scientific Instruments, Cranberry, PA, USA). The accelerating voltage for FE-SEM was 10 kV.

### 4.4. Release Kinetics of Aln from BCP Scaffold

To evaluate the *in vitro* release kinetics of Aln from Aln (1 mg)/BCP and Aln (5 mg)/BCP scaffolds, each sample was immersed in 1 mL of PBS buffer (pH 7.4) with gentle shaking (100 rpm) at 37 °C. At predetermined time intervals of one, three, five, and 10 h, and one, three, five, seven, 14, 21, and 28 days, the supernatants of the specimens were collected and replaced with an equal volume of fresh PBS solution. The samples were collected and stored at −20 °C prior to analysis. The absorbance of the Aln was determined using a Flash Multimode Reader (Varioskan™, Thermo Scientific, Waltham, MA, USA) at a wavelength of 293 nm with a complex of Aln and standard iron(III) chloride solution.

### 4.5. Osteogenic Differentiation Conditions

To demonstrate the enhancement of osteogenic differentiation on all scaffolds, alkaline phosphatase (ALP) activity and calcium content analyses were used. Prior to ALP activity, calcium content, and performing gene expressions analyses, all scaffolds were sterilized with 70% ethanol for 1 min and rinsed with PBS. Osteogenic differentiation medium (ODM) composed of DMEM supplemented with 10% FBS and 1% antibiotics in the presence of 50 μg/mL ascorbic acid, 10 nM dexamethasone, and 10 mM β-glycerophosphate, were used to evaluate ALP activity, calcium content, and gene expressions.

### 4.6. ALP Activity

MG-63 cells were seeded on BCP, Aln (1 mg)/BCP and Aln (5 mg)/BCP scaffolds in a 24-well tissue-culture plate at a concentration of 1 × 10^5^ cells/scaffold and incubated for up to 10 days in ODM. At predetermined time intervals of three, seven, and 10 days, the cells were lysed using 1× radioimmunoprecipitation assay buffer. The cell lysates were centrifuged at 13,500 rpm for 3 min at 4 °C. The supernatants were incubated with *p*-nitrophenyl phosphate solution for 30 min at 37 °C. The reaction was stopped by adding 500 μL of 1 N NaOH. ALP activity was determined by measuring the conversion of *p*-nitrophenyl phosphate to *p*-nitrophenol. Optical density was determined using a Flash Multimode Reader (Varioskan™) at a wavelength of 405 nm.

### 4.7. Calcium Content

MG-63 cells were seeded at a concentration of 1 × 10^5^ cells/mL on BCP, ALN (1 mg)/BCP, and ALN (5 mg)/BCP scaffolds in a 24-well tissue-culture plate. After 21 days of culture, 0.5 N HCl was added to the cells/scaffolds. Calcium deposition was measured in the supernatants using a QuantiChrom Calcium Assay Kit (DICA-500, BioAssay Systems, Hayward, CA, USA) according to the manufacturer’s instructions. Optical density was determined using a Flash Multimode Reader (Varioskan™) at a wavelength of 612 nm.

### 4.8. Gene Expression

To determine messenger RNA (mRNA) expression of osteogenic differentiation markers, such as osteocalcin (OCN) and osteopontin (OPN), we performed real-time polymerase chain reaction (PCR). MG-63 cells (1 × 10^5^ cells/mL) were seeded on BCP, Aln (1 mg)/BCP, and Aln (5 mg)/BCP scaffolds in a 24-well tissue-culture plate. After 21 days of culture, cDNA was synthesized with 1 μg total RNA and oligo (dT) primer using the Superscript First-Strand Synthesis System (Invitrogen, Carlsbad, CA, USA) according to the manufacturer’s instructions. The following oligonucleotide primers were used: OCN ((F) 5ʹ-TGA GAG CCC TCA CAC TCC TC-3ʹ, (R) 5ʹ-ACC TTT GCT GGA CTC TGC AC-3'); OPN ((F) 5ʹ-GAG GGC TTG GTT GTC AGC-3ʹ, (R) 5'-CAA TTC TCA TGG TAG TGA GTT TTC C-3'); glyceraldehyde 3-phosphate dehydrogenase (GAPDH) ((F) 5ʹ-ACT TTG TCA AGC TCA TTT CC-3', (R) 5'-TGC AGC GAA CTT TAT TGA TG-3'). Real-time PCR was performed with the above-mentioned specific primers. PCR amplification and detection were carried out on an ABI7300 Real-Time Thermal Cycler (Applied Biosystems, Foster, CA, USA) with the DyNAmo™ SYBR^®^ Green qPCR Kit (Finnzymes, Espoo, Finland). The relative mRNA expression levels of OCN and OPN were normalized to GAPDH expression.

### 4.9. Rat Tibial Defect and Treatment

Prior to the procedure of *in vivo* study using rat tibial defect model, our study were approved by the Institutional Animal Care and Use Committee of the Korea University Medical Center (KUIACUC-20130529-2). 24 Sprague-Dawley rats (Orient Bio Co., Seoul, Korea), aged eight weeks and weighing a mean 310 ± 14 g, were used in this study. Rats were divided into three groups as follows.

Group I: BCP only
Group II: Aln (1 mg)/BCP
Group III: Aln (5 mg)/BCP

**Figure 11:**
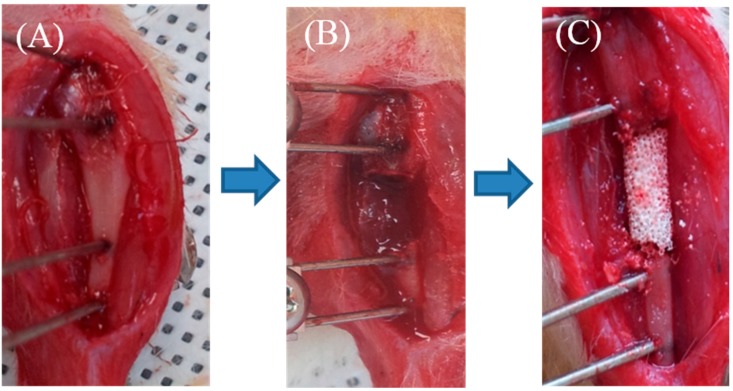
(**A**–**C**) Experimental animal model with a 7-mm-sized segmental diaphyseal tibial defect. (**A**) Rat’s tibia was exposed, and external fixator was applied; (**B**) 7mm sized segmental tibial defect was made; and (**C**) BCP scaffold of 7 mm length was inserted on the defect site.

Anesthesia was induced with an intraperitoneal injection of a commercial combination of tiletamine/zolazepam (143.75 mg/kg; Zoletil, Virbac Laboratories, Paris, France) and Xylazine (2.33 mg/kg; Rompun, Bayer Healthcare Korea, Seoul, Korea). The left leg of rat was shaved and prepared for sterile isolation. A 2 cm skin incision was made over the medial aspect of left tibia mid-shaft area. The periosteum and soft tissue carefully retracted to expose the tibia. Two 0.9 mm Kirschner-wires (K-wire) (Zimmer, Warsaw, IN, USA) were used to drill both tibial cortices. The K-wires were clamped bilaterally with custom-made external fixators. A 7-mm-sized diaphyseal defect was made using a cutting bur between two K-wires, and three different types of scaffold were applied according to each group. Subcutaneous tissue and skin was sutured (Figure 11). The animals were allowed free movement in cages after recovery from anesthesia. At two different time points (four and eight weeks), the progress of bone formation was evaluated with plain radiograph, micro-CT, Bone formation, and histology.

### 4.10. Analysis of Bone Formation

At four and eight weeks post operation, rats were sacrificed to evaluate bone formation by simple radiography and micro-CT. Rat tibiae were fixed a 3.7% paraformaldehyde solution. An X-ray apparatus (In vivo DXS 4000 Pro system, Carestream, Rochester, NY, USA) was used for rat tibiae simple radiograms. Bone formation was evaluated using a micro-CT scanning system (Albira II imaging system, Carestream). The CT system was operated at a voltage of 40 kV, and a of 250 μA current with a nominal resolution of 9 μm/pixel. Bone mineral density (BMD) and percentage bone volume (% BV) of defect site were obtained using micro-CT on four- and eight-week specimens.

### 4.11. Histological Evaluation

The rat tibiae/substrates were excised at four and eight weeks of post-operation period. They were fixed in 10% neutral buffered formalin, decalcified and embedded in paraffin. Tissue blocks were sectioned at 5 μm in thickness in the parallel longitudinal direction parallel and stained with hematoxylin and eosin (H and E) and Masson’s trichrome. Photographs of defect area were taken under 40× magnification and 200× magnification. New bone formation in defects was evaluated with a light microscope (CX31RTSF, Olympus, Tokyo, Japan).

### 4.12. Statistical Analysis

The mean and standard deviation of the values were computed and statistical analysis was performed using one-way ANOVA. Multiple comparison analysis was performed to test the difference between control and test groups. All statistics were verified at 95% significance level. Statistical significance was established at * *p* < 0.05 and ** *p* < 0.01. All statistical analyses were performed with PASW for Windows, version 18.0 (SPSS, Inc., Chicago, IL, USA).

## 5. Conclusions

In this study, sustained release of Aln from the Aln/BCP scaffolds was demonstrated with various concentrations. Aln/BCP scaffolds significantly enhanced the osteogenetic activity and mineralization by demonstrating a significant increase in ALP activity compared to activity in BCP scaffolds *in vitro*, and Aln/BCP scaffolds enhanced the osteogenetic effect in a rat tibia defect model *in vivo*. In conclusion, Aln/BCP scaffold demonstrated potential for improved sustained release of Aln, thus enhancing bone formation and mineralization. This scaffold could be a reasonable bone graft material for large bone defect in clinical situations.
